# Treatment of the Dental Pulp

**Published:** 1879-06

**Authors:** Arthur M. Rice


AMERICAN JOURNAL

OF

### DENTAL SCIENCE.

Vol. XIII. THIRD SERIES—JUNE, 1879. No. 2.

### ARTICLE I.

### *Treatment of the Dental Pulp.*

BY ARTHUR M. RICE, D. D. S.

The high degree of vitality existing in the dental pulp makes it subject to a variety of morbid changes, which are the result of circumstances, such as various temperaments, constitutional health, an abnormal condition of the hard structure of the tooth, attrition, erosion, &c. Sometimes much irritation is present in females during gestation without apparent organic change in any portion of the tooth structures or pulp, and dyspepsia and other functional disorders frequently produce the same effects.

But the diseases of the pulp are chiefly attributable to the presence of caries in the teeth, therefore, the most careful attention should be paid to a thorough examination and treatment of them, in order to prevent exposure and irritation of the lining membrane and preserve as nearly as possible the normal condition of the tooth. When the decay has made but little progress this is comparatively an easy operation, but unfortunately in very many cases it has progressed so far before being brought to our notice, that even to the most skillful the only course left open is to destroy sensibility by the entire destruction of the pulp, or, where there is a chance for success, to make use of the process termed capping.

In order to insure the best success in operations of this nature, one must be careful to consider the class to which a tooth belongs, the constitution, health and age of the patient, and the condition of the teeth and mouth.

Before describing the different methods of capping as a means of preserving the vitality of a tooth, it may be well to state a fact which is generally known to the profession, viz: a tooth may be decayed so nearly to the pulp, that the bone in direct contact with this part is in a softened condition, without causing an apparent inflammation or even great irritation of the pulp; and where a slight degree of irritation is present the amount of inflammation is not sufficient to cause pain. There may be considerable tenderness, and the pulp very near exposure, and still this organ be unaffected by disease. Now we know that in a large majority of cases after the human teeth have acquired their proper length, a deposition of bone from the pulp continues, and, in some cases, may eventually result in its entire obliteration. There are times when this deposition goes on more rapidly than at others, owing to various causes and conditions, (and the cases are by no means rare where decay threatens to expose the pulp,) and such a quantity is formed, as to arrest the further progress of the disease. This fact has no doubt been observed by all who have practiced any length of time, where a tooth having decayed to considerable extent, the decomposition seems to have been stopped in its course, and a healthy action been established, or at least there is no further breaking up of the parts, and the condition of the carious cavity remains, as it were, stationary. The reason why this action does not always take place can be explained by the fact that, in the majority of cases the caries progress so much more rapidly than the deposition of bone, that as a consequence the pulp is uncovered and is no longer able to perform this function. So good reasons exist for believing that if the progress of the caries can be arrested before arriving at this last stage, after a time a dense and healthy covering will be supplied to the pulp.

Now this theory is generally admitted to be the correct one, and the best practice is, in *all* cases where there is a probability of success, to carefully remove as much of the devitalized bone as possible without disturbing that portion directly over the pulp, which will serve as a covering; the next step is to syringe the cavity out carefully, and dry it, then apply a little creosote on a pellet of cotton, allowing it to remain about two minutes, remove this and fill the cavity with some non-irritant substance that will harden the decomposed dentine; "lacto-phosphate of lime is recommended as the best material to use;" this is covered with oxychloride of zinc sufficient to fill the cavity, allowing this to remain two or three months; a portion of it can then be removed. In order to test the success of the operation, if on examination we find vitality in the dentine, it is safe to infer the pulp is performing its healthy function. If during this period there has been no pain or uneasiness of the tooth, in nine cases out of ten we can fill with a permanent filling and feel assured of success. I have often followed a like course, and after the removal of the temporary filling have found the devitalized bone very hard and firm, and have been able to introduce a permanent filling of gold without inconvenience to the patient. The safest plan is to leave a slight covering of a non-irritant material, and fill over this to prevent thermal changes. This proves conclusively that, under favorable circumstances, by pursuing this method we arrive at most satisfactory results. Of course this treatment cannot be recommended in *all* cases, for, in certain conditions of the mouth and teeth such a course would only be attended by absolute failure, and the death of the pulp would surely follow. Although we cannot calculate with certainty the exact results of all operations of this kind, still a careful discrimination on the part of the operator will, in most cases, enable him to determine the proper method of treatment.

As a rule for all teeth belonging to class first, and in general those in which the devitalized bone is of a blackish or dark brown color and of rather hard consistency, the course of treatment described is attended by good results, but where the decay is of a soft, yielding nature and a light color, it is not advisable to attempt this course, especially if the tooth is one of much value.

We find some teeth that are almost worthless and of but little use to the patient, still he is loth to part with them and wishes them treated in some way. In these teeth we can try almost any experiment, as the result is not a matter of so much moment. After experience in all these matters, a practitioner's judgment will enable him to distinguish between those cases that would, in all probability prove a failure, and those upon which treatment would result in success.

After a series of experiments extending through many years, the conclusion reached by nearly all practitioners is, that a tooth can be preserved alive by the process termed capping, even after the lining membrane has become exposed. Now this has been performed in various ways, and it may be difficult to assert which method has been attended with the greatest degree of success, as each has had warm advocates who have claimed the best results. Of course each practitioner has his favorite method, and the diversity of opinion has led to investigations which have, without doubt been of great benefit to the profession generally.

One of the first methods practiced, consisted in carefully removing all the defective portion about the walls of the cavity and nicely fitting a gold cap over the pulp in such a manner that the edges should rest on the adjoining dentine so that there could be no injury to the pulp during the operation. It was necessary to exercise great care to prevent the displacement of the cap while introducing the filling, and many times this proved a very difficult matter. Under some circumstances this method might prove desirable and no doubt a few operators have met with success in its use, but owing to the nicety of the operation it has been superseded by others more practicable and less difficult.

A method very similar to this one is employed by a dentist of acknowledged ability and one regarded as a fine operator. This consists in fitting a piece of bone or ivory to the bottom of a cavity, and where it comes in contact with the pulp he makes the cap slightly concave in form so that there shall be no pressure on this organ, and then proceeds to fill the tooth. Of course the same care must be exercised as in the first instance to prevent any change in the position of the cap, and where there is great difficulty in doing this he has an assistant to hold it in position until it is secured in the process of packing the gold. There are some locations and conditions of a tooth where this method could not be employed, yet in a great many cases he claims success for it.

Another method successfully adopted in the treatment of exposed pulps by a dentist of considerable note, consists in covering it with a paste composed of plaster of Paris and the oil of cloves. In preparing the cavity he is careful to remove all particles of decomposed matter, and then to touch the exposed surface with creosote largely diluted before applying the paste; after this has become sufficiently hard the filling is introduced, and (according to his own statement) by following this conrse he is almost universally successful.

The most common method and one which is now almost wholly adopted by the profession is the use of some Oxychloride of Zinc preparation in all cases where capping is necessary. When these combined agents oxide and chlorine of zinc were first introduced as a material for capping, there were many cautious operators who gradually and with great care adopted its use, feeling their way cautiously at each step, and they were slow to confess its merits or declare the result of their experiments, only trying it in the most favorable cases. On the other hand may unskillful and careless ones at once employed it in *all* cases, even where grave doubts existed as to success, and as a consequence in a great many instances it was effectual in bringing about the very results that they were seeking to avoid. It was the custom to apply it directly to the pulp in its full strength, and the strong escharotic property of the chloride made itself manifest, first by the extreme pain produced, and after a time by the complete destruction of the pulp; but by carefully observing the results of repeated experiments we have learned how to avoid this mistake.

To use oxychloride of zinc successfully considerable care must be exercised. The material should be pure and properly prepared. The oxide of zinc is often impure, containing white lead, chalk, and other substances, and the yellowish white color is regarded as indicating the best quality. The chloride should not be used in its full strength, as far better results are assured with a diluted solution only sufficiently strong to cause the mixture to set. This can be diluted by adding a little water, or what is better a little chloroform, the anodyne effects of which will have a tendency to allay the pain produced by the chloride.

In the case of partially exposed pulps and where there is a degree of irritation present, the first step would be to remove all extraneous matter, then apply the rubber dam and dry carefully, and if any portion of the soft devitalized part remained, remove this with round edged excavators, then an alkaline solution, as bicarbonate of soda, can be applied which will leave a tendency to relieve any pain that may be present. A solution of gutta-percha in chloroform is now placed over the bottom of the cavity, and after the chloroform has evaporated, the oxychloride is used to cover this or to fill the cavity temporarily.

In cases of wholly exposed pulps where there is no inflammation present, and also where they are exposed during the process of preparing a cavity, as they often are, slight hemorrhage follows, but this usually comes from the veins which are superficial, and but little difficulty attends the preservation of the pulp, but where the arteries are wounded the case becomes more complicated and success does not so easily follow our treatment. By the aid of a magnifying glass, we are enabled to distinguish the exposure of an artery by its pulsations. Now the dental pulp is subject to the same conditions as other soft tissues to a certain extent, and our diagnosis should be a careful one in order to ascertain with accuracy just how far it is involved in disease, and it is a very important point to determine the difference between normal and abnormal conditions. The pulp in a normal state is not very sensitive, and we often come upon the cornua of this organ, which extends into the cavity of a tooth without causing much pain; but where inflammation is present, a breath of air even will cause the patient to start and show signs of suffering.

In the case of simple exposure and slight hemorrhage attending it, we must wipe the cavity out and stop the blood; this can be done by using tinc. of calendula or spirits of camphor. After we have accomplished this, the part is then touched with glycerine, as a soothing agent. We now proceed to apply our collodion, or gutta-percha in chloroform, which is covered with oxychloride as in the preceding cases; after this has become sufficiently hard the surplus can be removed, and a gold filling introduced. Of course it would be better in *all* cases to wait a few months before filling permanently, although many operators are in the habit of finishing the operation at one sitting. Now the advantages of waiting are these—we cannot always be sure of good results, especially where there has been some irritation of the lining membrane and pain in the tooth, and by this precaution some trouble to ourselves and annoyance to our patients may be saved; another advantage is that as the temporary filling wears out we can test the success of the operation by cutting a portion of the dentine; if this retains its vitality, we are assured that the pulp is in a normal condition. This deposition of secondary dentine goes on so slowly in the majority of cases, that a year or more will be necessary to the building up of a new covering to the pulp. Of course this depends somewhat on the health of the patient, the condition of the blood, &c. Sometimes we know that slight inflammation may exist soon after capping, which passes away and no further uneasiness is felt, but experience shows cases like these to be exceptions, and consequently, we must endeavor to prevent irritation that may lead to inflammation of the membrane.

[TO BE CONTINUED.]

